# Elevated Neddylation Pathway Promotes Th2 Cells Infiltration by Transactivating STAT5A in Hepatocellular Carcinoma

**DOI:** 10.3389/fonc.2021.709170

**Published:** 2021-11-05

**Authors:** Lisha Zhou, Luyi Zhang, Siyuan Chen, Dongsheng Sun, Jianhua Qu

**Affiliations:** ^1^ Department of Basic Medical Science, Medical College, Taizhou University, Taizhou, China; ^2^ Department of Hepatobiliary Surgery, Peking University Shenzhen Hospital, Shenzhen, China

**Keywords:** neddylation, Th2 cells, STAT5A, hepatocellular carcinoma, immune response

## Abstract

Neddylation is a process in which a ubiquitin-like molecule NEDD8 is conjugated to a lysine residue of the substrate protein *via* successive enzymatic cascade reactions. Inactivation of neddylation pathway triggers tumor cell apoptosis or senescence to suppress the tumor growth. So far, there has been limited research on the role of the neddylation pathway (NEDD8-UBE2M-RBX1 axis) in the immune response. In this study, we investigated the association between the neddylation pathway and immune function in HCC by comprehensively analyzing transcriptome and clinical data of HCC samples from TCGA database. The analysis showed that the mRNA expression of neddylation pathway components was up-regulated in HCC and increased with disease severity. Moreover, we observed that activated neddylation pathway was associated with enriched infiltration of T helper 2 (Th2) cells in HCC, while transactivation of STAT5A signaling may mediate this association. On the contrary, no significant correlation between the neddylation pathway and Th1 cells infiltration was identified. Taken together, these findings suggest a potential role of the neddylation pathway in promoting a shift in Th1/Th2 balance toward Th2-dominant immunosuppression. Hence, targeting neddylation pathway could serve as an attractive immunotherapy strategy for suppressing the development of Th2 cells.

## Introduction

Hepatocellular carcinoma (HCC) is the most common type of primary liver cancer and the third most frequent cause of cancer-related death worldwide (1). Due to the high rate of recurrence and metastasis, the five-year survival rate for advanced HCC is very low (2). As an immunogenic tumor, HCC is characterized by strong immunosuppressive microenvironment and high immune evasion (3). Immunotherapy is designed to harness the immune system to attack tumor cells, for reducing the rate of tumor recurrence and metastasis. As a result, immunotherapy has gradually become a promising direction for the treatment of advanced HCC (4, 5). However, only a small number of cancer patients could benefit from current immunotherapies. It is urgent to elucidate the regulatory mechanisms of immune cells infiltration in HCC and identify novel immune-related therapeutic targets.

CD4^+^ helper T (Th) cells are critical regulators of tumor immunity. The cells are mainly divided into two distinct subsets based on cytokines production and immunologic roles: Th1 and Th2 cells (6). Th1 cells produce characteristic cytokines interleukin (IL)-2, interferon (IFN)-γ and tumor necrosis factor (TNF)-α, which primarily mediate anti-tumor immunity and are associated with good prognosis in HCC patients. In the meantime, Th2 cells secret IL-4 and IL-10, facilitating tumor growth or metastasis *via* immunosuppression (7, 8). Among the cytokines produced by Th2 cells, IL-4 inhibits the secretion of IFN-γ by Th1 cells (9), and IL-10 suppresses the proliferation of Th1 cells as well as IFN-γ production (10, 11). Th1/Th2 imbalance has been observed in HCC patients with an elevation of Th2-released cytokines (12). Therefore, development of new strategies to alleviate Th2 cells infiltration may potentially contribute to HCC treatment.

Neddylation is a process in which a ubiquitin-like molecule neuronal precursor cell-expressed developmentally down-regulated protein 8 (NEDD8) is conjugated to a lysine residue of the substrate protein, *via* successive enzymatic cascade reactions catalyzed by NEDD8-activating enzyme E1 (NAE, a heterodimer of NAE1 and UBA3), NEDD8-conjuagating enzyme E2 (UBE2M or UBE2F) and substrate-specific NEDD8-E3 ligase (RBX1 and RBX2, ect) (13–15). The best-characterized physiological substrates of neddylation are the cullin family members, subunits of Cullin-RING ligases (CRLs) (16, 17). While NEDD8-conjuagating enzyme E2 UBE2M interacts with ring-box 1 (RBX1) to catalyze the neddylation of CUL-1, -2, -3, -4A and -4B, whereas E2 UBE2F specifically pairs with RBX2 to promote CUL-5 neddylation and activation (18, 19). Numerous studies have shown that inhibition of neddylation pathway leads to inactivation of CRLs and the accumulation of various CRL substrates, resulting in apoptosis or senescence of tumor cells (14, 15). MLN4924, a first-in-class inhibitor of NAE (20), which has entered phase I/II/III clinical trials in patients with solid tumors or hematological malignancies, shows potent anti-tumor activity and well-tolerated toxicity (14, 15). All these data highlight the neddylation pathway as a promising anticancer target.

We previously showed that, in addition to directly targeting tumor cells, neddylation inactivation suppresses the infiltration of myeloid-derived suppressor cells (MDSCs)/tumor-associated tumors (TAMs) into tumor sites to foster an immunosuppressive microenvironment, thus inhibiting the tumor growth (21, 22). To date, studies on the role of neddylation pathway in other tumor immune response such as Th1/Th2 balance are still lacking. To further validate the neddylation pathway as a promising anti-tumor target, this study investigated the relationship between activated neddylation pathway (NEDD8-UBE2M-RBX1 axis) and immune cell infiltration by analyzing mRNA transcriptome and clinical data of HCC samples from The Cancer Genome Atlas (TCGA) database.

## Materials and Methods

### Data Collection

The mRNA transcriptome data in TPM format and clinical information of HCC samples were obtained from the The Cancer Genome Atlas (TCGA) database (https://portal.gdc.cancer.gov/). A total of 424 samples including 50 normal tissues and 374 HCC tumor tissues were used for follow-up research. The samples were divided into the high expression group and low expression group according to the median value of the target protein expression level. The detailed clinicopathological characteristics of HCC patients were described in **Supplementary Table S1**.

The protein expression datasets and clinical information (160 patients; female, n=32; male, n=128) of cancer used for the analyses described in this study were obtained from the Clinical Proteomic Tumor Analysis Consortium (CPTAC) database (https://cptac-data-portal.georgetown.edu/datasets).

### ROC Curve Analysis and Kaplan Meier Survival Curve Analysis

The receiver operator characteristic (ROC) curve was used to determine the diagnosis efficacy of each factor (R package pROC). Kaplan-Meier analyses were compared using the log-rank test. Survival and survminer packages were used for Kaplan-Meier curves in R language. Survival package was used for computing survival analyses. Survminer package was used for summarizing and visualizing the results of survival analyses.

### Immune Infiltration Analysis

The single-sample Gene Set Enrichment Analysis (ssGSEA) was performed to quantify the infiltration levels of immune cells by using GSVA package (23). The correlation of neddylation pathway components or STAT5A/B with the immune cell infiltration was examined by Spearman’s correlation analysis.

### Cell Culture and Reagents

Human hepatocellular carcinoma cell line Huh7 was obtained from the National Collection of Authenticated Cell Cultures and passaged five to six times before use. Cells were cultured in Dulbecco’s modified Eagle’s medium (Gbico) supplemented with 10% fetal bovine serum (Gbico) and 1% penicillin-streptomycin solution (Gbico) at 37°C in 5% carbon dioxide. MLN4924 was dissolved in dimethyl sulfoxide (DMSO) and kept at -20°C for *in vitro* studies.

### Cell Proliferation

Cells were seeded in 96-well plates with 5000 cells per well in triplicate. Cell proliferation was analyzed using a Cell Counting Kit-8 (CCK-8, Dojindo Laboratories) according to the manufacturer’s recommendation.

### RNA Isolation and Real-Time PCR

Total RNA was isolated using Trizol reagent (Invitrogen) and treated with RNase-free DNase according to the manufacturer’s instructions. Reverse transcription was performed on 1 µg of total RNA per sample using the PrimerScript reverse transcription reagent kit (TaKaRa). The real-time polymerase chain reaction (PCR) was carried out using the TB Green Premix Ex Taq (TaKaRa) on the ABI Step One (Applied Biosystems). The mRNA abundance in each sample was normalized to the amount of actin. The sequences of the primers are as follows:

Human β-actin: forward 5′-TGACGTGGACATCCGCAAAG-3′,
reverse 5′-CTGGAAGGTGGACAGCGAGG-3′;
Human STAT5A: forward 5′-GCAGAGTCCGTGACAGAGG-3′,
reverse 5′-CCACAGGTAGGGACAGAGTCT-3′;

### Western Blotting

Cells were lysed in SDS sample buffer and denatured by heating on 100°C for 10 minutes. Proteins were separated on 10% Bis-Tris polyacrylamide gels. Western blot images were captured by Amersham Imager 680 (GE Healthcare). Antibody for STAT5A (ab32043) was purchased from Abcam. β-actin (M1210-2) was purchased from HuaBio.

### Statistical Analysis

R 3.6.3 was used for bioinformatic analysis. Spearman’s correlation analysis was performed to assess the potential correlations. Kaplan-Meier method and Log-rank test were used to determine the Overall Survival rate and the differences between groups, respectively. Statistical analysis of Cell proliferation and RT-PCR was conducted by using GraphPad Prism. P value < 0.05 was considered statistically significant.

## Results

### Expression of Neddylation Pathway Components and Its Prognostic Significance

**Figure 1:**
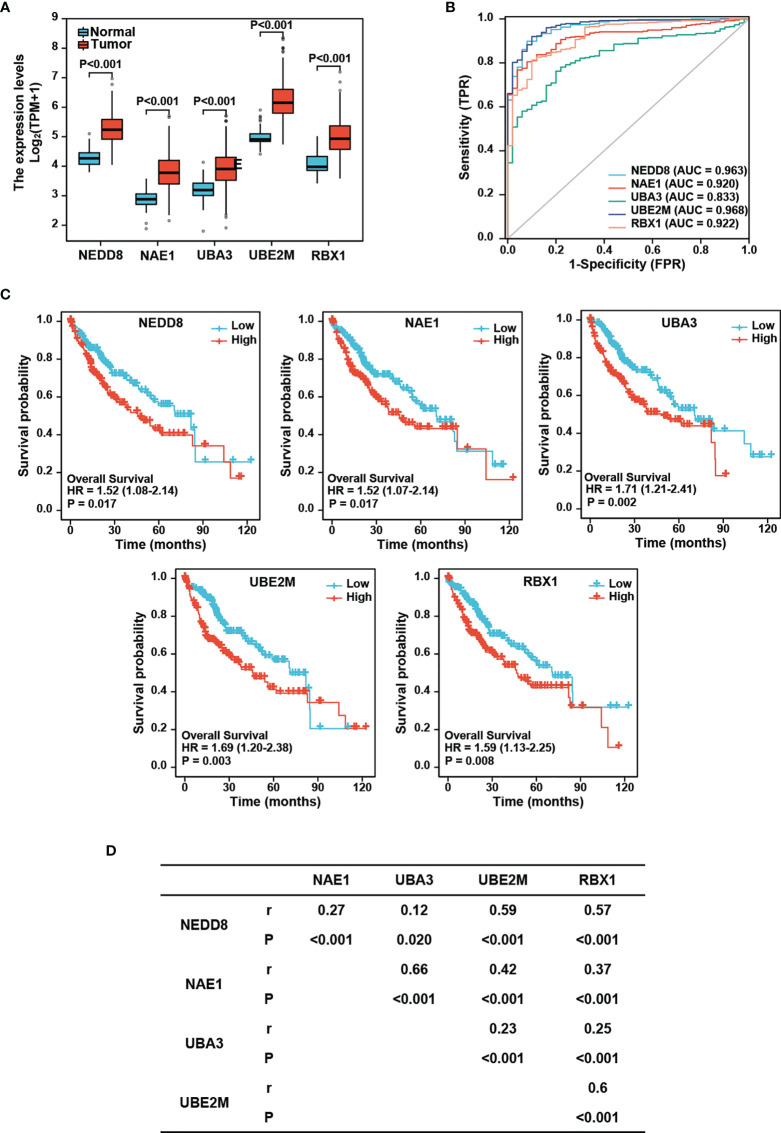
Expression of neddylation pathway components and the prognostic significance in HCC. **(A)** mRNA levels of neddylation pathway components are higher in hepatocellular carcinoma tissues compared with normal liver tissues. The RNAseq data in TPM form and clinical information were obtained from TCGA (https://portal.gdc.cancer.gov/) LIHC data. **(B)** ROC curve analysis of neddylation pathway components. **(C)** High expression of neddylation pathway components is associated with worse prognosis. **(D)** Neddylation pathway components positively correlate with each other.

To investigate the role of the neddylation pathway (NEDD8-UBE2M-RBX1 axis) in the occurrence and development of HCC, we comparatively analyzed the expression profile of major neddylation pathway components between normal liver tissues (n=50) and HCC tissues (n=374). The RNAseq data in TPM form and clinical information were obtained from the TCGA-LIHC dataset (https://portal.gdc.cancer.gov/). As shown in **Figure 1A**, a significant increase in the mRNA expression of NEDD8, NEDD8-activating enzyme E1 (NAE1 and UBA3), NEDD8-conjuagating enzyme E2 (UBE2M), or NEDD8-E3 ligase (RBX1) was detected in HCC tissues compared with normal liver tissues. Moreover, protein levels of all five neddylation pathway components in HCC group were higher than those in normal group based on CPTAC data (**Supplementary Figure S1A**). Meanwhile, ROC curve analysis revealed that all the five neddylation pathway components, especially UBE2M, had a good diagnostic value for HCC (AUC: NEDD8 = 0.963, NAE1 = 0.920, UBA3 = 0.833, UBE2M=0.968, and RBX1 = 0.922) (**Figure 1B**). These results demonstrate that the neddylation pathway components are transcriptionally up-regulated in HCC and may serve as potential diagnostic markers.

We next performed Kaplan-Meier analysis and found that HCC patients with high expression of NEDD8, NAE1, UBE2M, or RBX1 displayed a lower overall survival rate than those with low expression of the corresponding protein (Log-rank test: NEDD8, P=0.017; NAE1, P=0.017; UBA3, P=0.002; UBE2M, P=0.003; and RBX1, P=0.008) (**Figure 1C**). In CPTAC database, only the patients with high protein levels of UBA3 or UBE2M conferred poorer overall survival than those with low expression in HCC patients (Log-rank test: UBA3, P=0.029; UBE2M, P=0.037); (**Supplementary Figure S1B**). Together, these findings indicate that among these components, UBE2M possesses better prognostic significance for HCC.

Given that neddylation involves successive enzymatic cascade reactions catalyzed by the above-mentioned components, we further examined the correlations of these components with each other in the expression levels. Spearman’s correlation analysis revealed that the neddylation pathway components were significantly positively correlated with each other in HCC (r=0.12-0.66, P=0.02 to P<0.001) (**Figure 1D**), suggesting that these components appear to be coordinately regulated.

### Neddylation Pathway Is Associated With Th2 Cells Infiltration in HCC

**Figure 2:**
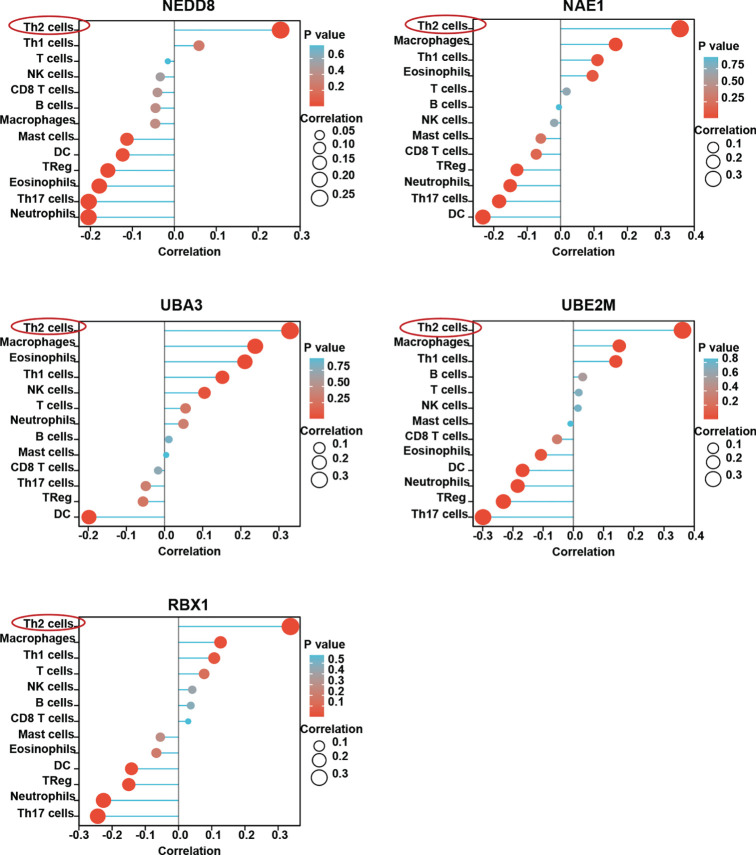
Neddylation pathway is closely related with immune cells infiltration in HCC. ssGSEA was used to evaluate the correlation of neddylation pathway and 13 immune infiltration cells in HCC.

HCC is considered an immunogenic tumor with strong immunosuppressive microenvironment and high immune evasion (3). We therefore sought to determine the relationship between the neddylation pathway and tumor immune microenvironment in HCC. As depicted in **Figure 2**, we observed that the neddylation pathway components, in varying degrees, were negatively correlated with the infiltration of DCs and Th17 cells, indicators for anti-tumor function. Notably, the infiltration of Th2 cells in HCC displayed the most significant positive correlation with all the components (**Figure 2**). These observations suggest that the neddylation pathway may play an important role in the immune response in HCC.

**Figure 3:**
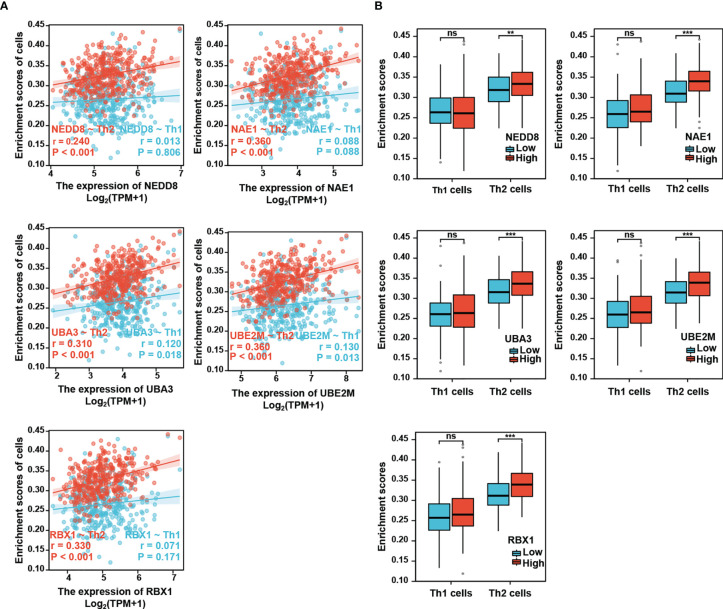
Neddylation pathway positively correlates with the infiltration of Th2 cells, but not Th1 cells. **(A)** Neddylation pathway components positively correlate with the infiltration of Th2 cells, but not Th1 cells. **(B)** The infiltration of Th2 cells, but not Th1 cells, is significantly increased in the activated neddylation pathway group. **P<0.01, ***P<0.001, ns, not significant, for the indicated comparison.

It has been shown that while Th1 cells mediate anti-tumor effect, Th2 cells contribute to favor tumor growth (7, 24). Th1/Th2 imbalance has been observed in HCC patients with down-regulation of Th1-released cytokines and up-regulation of Th2-released cytokines (12). Next, we further investigated the relationship of the neddylation pathway with Th1/Th2 balance. The analysis showed that compared with Th1 cells, Th2 cells were much more significantly positively correlated with the neddylation pathway (NEDD8-Th2: r=0.24, p<0.001; NAE1-Th2: r=0.36, p<0.001; UBA3-Th2: r=0.31, p<0.001; UBE2M-Th2: r=0.36, p<0.001; RBX1-Th2: r=0.33, p<0.001) (**Figure 3A**). Moreover, a significant increase in the infiltration level of Th2 cells, rather than Th1 cells, was identified in high expression group of neddylation pathway components as compared to the low expression group (**Figure 3B**). All these data reveal a potential role of the neddylation pathway in promoting a shift in Th1/Th2 balance toward Th2-dominant immunosuppression.

**Figure 4:**
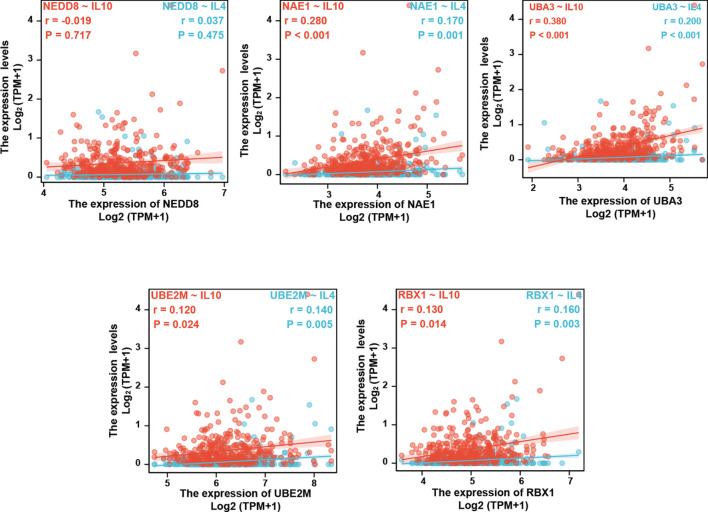
Neddylation pathway positively correlates with Th2-released cytokines IL-4/IL-10.

To further verify this result, we examined whether the neddylation pathway is associated with Th2-released cytokines IL-4/IL-10. Clearly, all tested pathway components except NEDD8 (NAE1, UBA3, UBE2M, and RBX1) were positively correlated with IL-4 and IL-10 expression, as indicated by Spearman’s correlation analysis (**Figure 4**). Based on these findings, we reasoned that the neddylation pathway may participate in the development of Th2 cells.

### Neddylation Pathway Transactivates STAT5A

**Figure 5:**
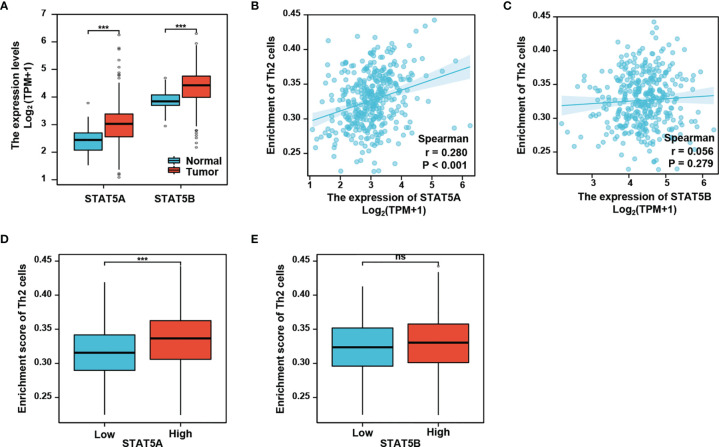
STAT5A, but not STAT5B, is associated with Th2 cells infiltration. **(A)** STAT5A and STAT5B are significantly up-regulated in HCC compared with normal liver tissues. **(B, C)** Th2 cells infiltration in HCC is positively correlated with STAT5A, but not STAT5B. **(D, E)** Th2 cells infiltration is increased in STAT5A high group, but not in STAT5B high group. ***P<0.001, ns, not significant, for the indicated comparison.

Given that activation of signal transducer and activator of transcription 5 (STAT5) is critical for Th2 cell differentiation (25), we then explored a possible role of STAT5 in linking the neddylation pathway to Th2 cell infiltration in HCC. As depicted in **Figure 5A**, both STAT5A and STAT5B, the two isoforms of STAT5 were significantly up-regulated in HCC tissues as compared to normal liver tissues (P<0.001). This observation prompted us to determine that whether STAT5A/5B are correlated with Th2 cell infiltration in HCC. The study showed that the infiltration of Th2 cells in HCC was significantly positively correlated with STAT5A (P<0.001), rather than with STAT5B (P>0.05) (**Figures 5B, C**). Moreover, the infiltration of Th2 cells in high expression group of STAT5A was significantly higher than that in the low expression group (**Figure 5D**). On the contrary, no significant correlation between STAT5B expression and the filtration of Th2 cells was identified (**Figure 5E**).

**Figure 6:**
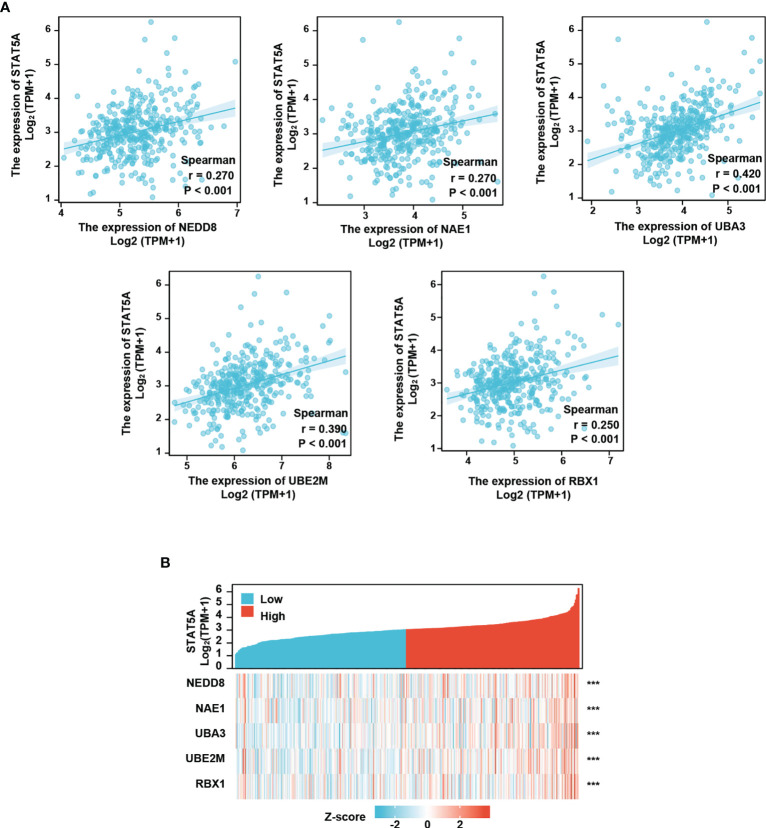
Neddylation pathway positively correlates with STAT5A. **(A)** Neddylation pathway components positively correlate with STAT5A. **(B)** Neddylation pathway components are increased in STAT5A high group. ***P<0.001, for the indicated comparison.

We next conducted Spearman’s correlation analysis to further analyze the correlation between the neddylation pathway and STAT5A/5B. As shown, STAT5A significantly positively correlated with all the pathway components (STAT5A- NEDD8: r=0.27, p<0.001; STAT5A-NAE1: r=0.27, p<0.001; STAT5A-UBA3: r=0.42, p<0.001; STAT5A-UBE2M: r=0.39, p<0.001; STAT5A-RBX1: r=0.25, p<0.001) (**Figure 6A**); While, STAT5B only correlated with NAE1 and UBA3 (STAT5B-NAE1: r=0.40, P<0.001; STAT5B-UBA3: r=0.58, P<0.001) (**Supplementary Figure S2**). To assess the co-expression of STAT5A and neddylation pathway components in HCC, we performed the heatmap analysis on both STAT5A low expression- and high expression groups. The analysis revealed significant up-regulation of these components in STAT5A high expression group compared with the low expression group (P<0.001) (**Figure 6B**).

**Figure 7:**
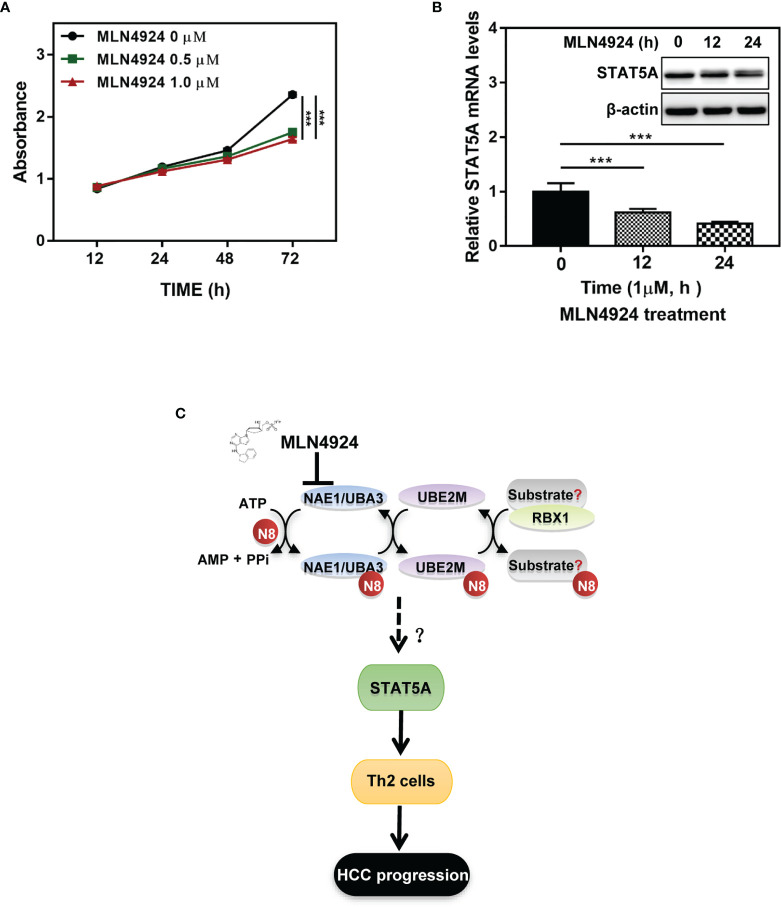
Inactivation of neddylation pathway suppresses the transcription of STAT5A in HCC cells. **(A)** MLN4924 treatment significantly inhibited the proliferation of hepatocellular carcinoma cell line Huh7. **(B)** MLN4924 treatment decreases STAT5A mRNA and protein levels. Huh7 cells were treated with MLN4924 in a time-dependent manner as indicated. **(C)** Shown is a working model depicting the potential mechanism of neddylation pathway involved in the development of Th2 cells to favor tumor progression. ***P<0.001, for the indicated comparison.

As a potent and selective inhibitor of NAE, MLN4924 blocks protein neddylation by inactivating the initial step of neddylation cascade, and exerts significant anticancer effects by inhibiting cell proliferation (14, 20). Consistent with previous studies, MLN4924 treatment significantly inhibited the proliferation of hepatocellular carcinoma cell line Huh7 (**Figure 7A**). Then we further characterized the role of STAT5A in mediating the neddylation pathway in HCC. Clearly, MLN4924 treatment led to a significant time-dependent decrease in both the mRNA and protein levels of STAT5A (**Figure 7B**). These data suggest that the neddylation pathway may promote the development of Th2 cells by transactivating STAT5A.

## Discussion

HCC is considered an immunogenic tumor with a strong immune suppressive microenvironment as well as a high immune evasion (3). In this study, we analyzed mRNA sequencing data of HCC samples that were collected from the TCGA database and identified a significant correlation between the neddylation pathway (NEDD8-UBE2M-RBX1 axis) and Th2 cells infiltration in HCC. Hence, this study provided novel insights into the role of the neddylation pathway in Th2 cell-mediated tumor immunosuppression (**Figure 7C**).

Extensive studies have shown that while the neddylation pathway components are abnormally activated in multiple types of cancers, the pathway inhibition can trigger cancer cell apoptosis or senescence to significantly suppress the tumor growth (14, 15). These studies developed sound rationale for targeting the neddylation pathway as an attractive anti-tumor therapeutic strategy. However, there has been limited research on the role of the neddylation pathway in the immune response. In the previous studies, we found that, in addition to targeting cancer cells, neddylation inactivation can inhibit the infiltration of myeloid-derived suppressor cells (MDSCs)/tumor-associated tumors (TAMs) into the tumor sites to foster an immunosuppressive microenvironment, thereby suppressing the tumor growth (21, 22). Here, we investigated the association between the neddylation pathway and immune function in HCC by comprehensively analyzing transcriptome and clinical data of HCC samples from TCGA database. The present study showed that the infiltration level of Th2 cells rather than Th1 cells was significantly increased in high expression group of neddylation pathway as compared to the low expression group. Moreover, we observed that Th2-cytokines such as IL-4 and IL-10 were more significantly expressed in the high expression group of neddylation pathway. Collectively, these findings suggest a potential role of the neddylation pathway in promoting a shift in Th1/Th2 balance toward Th2 cells. Naive T lymphocytes (Th0) can differentiate into Th1 and Th2 cells which exert anti- and pro-tumor effects, respectively. It has been shown that the Th1/Th2 balance is disturbed in HCC patients with down-regulation of Th1-released cytokines and up-regulation of Th2-released cytokines (12). In the present study, we provided more evidence that targeting neddylation pathway could be an attractive immunotherapy regimen for suppressing the shift from Th1 to Th2 cells.

We further analyzed the expression of key factors in the pathway of Th2 differentiation to investigate mechanisms underlying the role of the neddylation pathway in Th2 cell development. While activated STAT5 has been found to play an irreplaceable role in differentiation of Th0 cells into Th2 cells (25), the activation of STAT5 is dependent on its posttranslational modifications, including phosphorylation, acetylation, SUMOylation, and ubiquitination (26). In the present study, the analysis on the RNAseq data revealed that the neddylation pathway positively correlated with STAT5A, the most dominant isoform of STAT5 capable of inducing the expression of downstream IL-4. Moreover, we found that MLN4924-induced inactivation of neddylation pathway significantly inhibited the transcriptional expression of STAT5A *in vitro*. Together, these results imply that the transcriptional activation of STAT5A might be involved in the regulation of Th2 cell development by the neddylation pathway. Interestingly, MLN4924 treatment resulted in weak band-shift of STAT5A, which merits further investigation.

There are still some limitations in this study. First, while we identified a positive correlation between the neddylation pathway and Th2 cell infiltration in HCC, this finding needs to be further characterized in the tumor-bearing mouse model by using specific pharmacologic agents or genetically targeting the neddylation pathway. Second, neddylation pathway-regulated transcriptional expression of STAT5A awaits further investigation both *in vitro* and *in vivo*.

In conclusion, this study presents evidence that activated neddylation pathway is associated with enriched Th2 cell infiltration in HCC, while the transactivation of STAT5A signaling may mediate the association. Meanwhile, we showed that neddylation pathway exerts obvious effects on HCC patients’ clinical outcome, suggesting a critical role of the pathway in HCC progression. Hence, targeting neddylation pathway for inhibition of Th2 cell development could serve as a potentially attractive anti-tumor immunotherapy strategy.

## Data Availability Statement

The original contributions presented in the study are included in the article/**Supplementary Material**. Further inquiries can be directed to the corresponding authors.

## Author Contributions

LSZ, LYZ, SC, DS, and JQ were involved in study conceptualization, methodology, data collection and analysis. LSZ, DS, and JQ were involved in manuscript writing and reviewing. All authors contributed to the article and approved the submitted version.

## Funding

This work was supported by the National Natural Science Foundation of China (Grant Nos. 81702244, 81871870, and 82073069), and Zhejiang Provincial Natural Science Foundation of China (Grant No. LY21H160008).

## Conflict of Interest

The authors declare that the research was conducted in the absence of any commercial or financial relationships that could be construed as a potential conflict of interest.

## Publisher’s Note

All claims expressed in this article are solely those of the authors and do not necessarily represent those of their affiliated organizations, or those of the publisher, the editors and the reviewers. Any product that may be evaluated in this article, or claim that may be made by its manufacturer, is not guaranteed or endorsed by the publisher.
