# Supplementary material for: Elevated Neddylation Pathway Promotes Th2 Cells Infiltration by Transactivating STAT5A in Hepatocellular Carcinoma
Source: Front Oncol. 2021 Nov 5;11:709170. doi: 10.3389/fonc.2021.709170 (PMC8602568; doi:10.3389/fonc.2021.709170)
Supplement: Supplementary file 1 [file DataSheet_1.docx]

**Supplementary Figure S1**

**A**

**
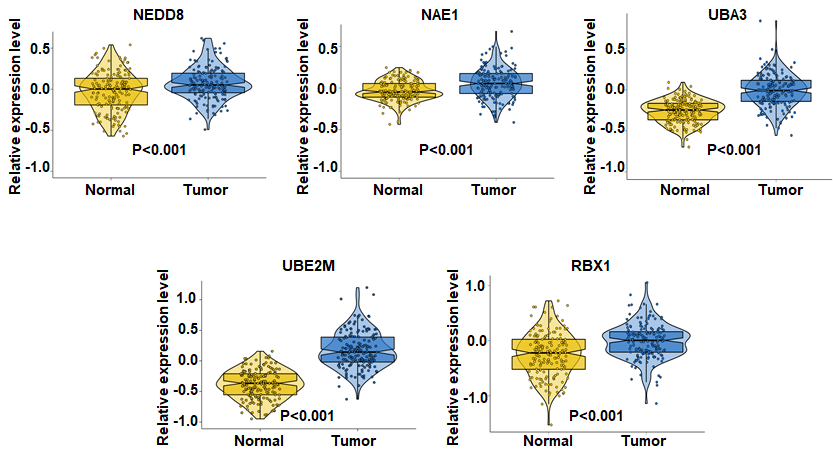
**

**B**

**
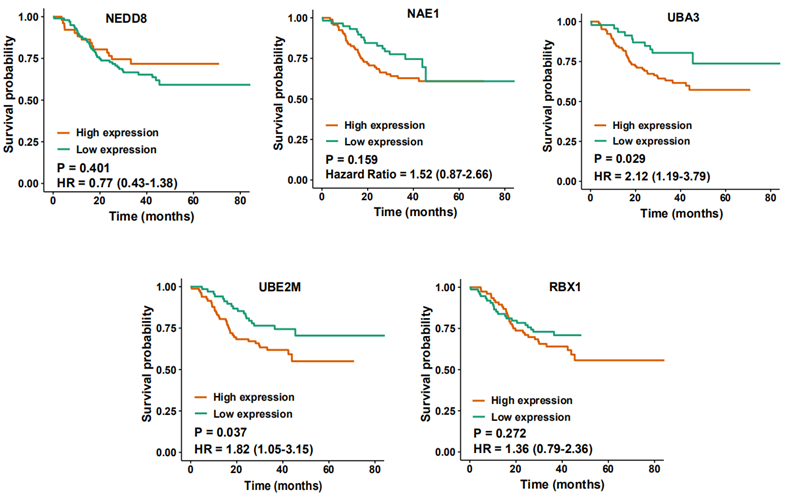
**

**Supplementary Fig S1. The expression datasets and clinical information were obtained from CPTAC (https://cptac-data-portal.georgetown.edu/datasets).**

**Supplementary Figure S2**

**
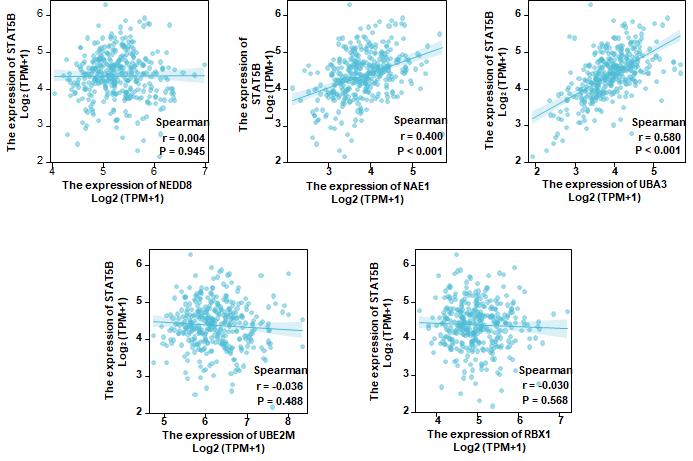
**

**Supplementary Fig S2. The correlation between neddylation pathway components and STAT5B.**

**Supplementary Table S1. Clinicopathologic parameters according to the expression of NEDD8, NAE1, UBA3, UBE2M and RBX1**

| **Characteristic** | **NEDD8** | | ***p*** | **NAE1** | | ***p*** | **UBA3** | | ***p*** | **UBE2M** | | ***p*** | **RBX1** | | ***p*** |
| --- | --- | --- | --- | --- | --- | --- | --- | --- | --- | --- | --- | --- | --- | --- | --- |
|  | **Low** | **High** |  | **Low** | **High** |  | **Low** | **High** |  | **Low** | **High** |  | **Low** | **High** |  |
| **T stage, n (%)** |  |  | 0.081 |  |  | 0.135 |  |  | 0.251 |  |  | **0.013** |  |  | **0.014** |
| **T1** | 100 (27%) | 83 (22.4%) |  | 98 (26.4%) | 85 (22.9%) |  | 97 (26.1%) | 86 (23.2%) |  | 107 (28.8%) | 76 (20.5%) |  | 106 (28.6%) | 77 (20.8%) |  |
| **T2** | 39 (10.5%) | 56 (15.1%) |  | 50 (13.5%) | 45 (12.1%) |  | 50 (13.5%) | 45 (12.1%) |  | 39 (10.5%) | 56 (15.1%) |  | 45 (12.1%) | 50 (13.5%) |  |
| **T3** | 42 (11.3%) | 38 (10.2%) |  | 33 (8.9%) | 47 (12.7%) |  | 35 (9.4%) | 45 (12.1%) |  | 33 (8.9%) | 47 (12.7%) |  | 30 (8.1%) | 50 (13.5%) |  |
| **T4** | 4 (1.1%) | 9 (2.4%) |  | 4 (1.1%) | 9 (2.4%) |  | 4 (1.1%) | 9 (2.4%) |  | 6 (1.6%) | 7 (1.9%) |  | 5 (1.3%) | 8 (2.2%) |  |
| **N stage, n (%)** |  |  | 0.624 |  |  | 0.623 |  |  | 0.622 |  |  | 0.623 |  |  | 0.622 |
| **N0** | 122 (47.3%) | 132 (51.2%) |  | 124 (48.1%) | 130 (50.4%) |  | 125 (48.4%) | 129 (50%) |  | 123 (47.7%) | 131 (50.8%) |  | 125 (48.4%) | 129 (50%) |  |
| **N1** | 1 (0.4%) | 3 (1.2%) |  | 1 (0.4%) | 3 (1.2%) |  | 1 (0.4%) | 3 (1.2%) |  | 1 (0.4%) | 3 (1.2%) |  | 1 (0.4%) | 3 (1.2%) |  |
| **M stage, n (%)** |  |  | 0.624 |  |  | 1.000 |  |  | 0.623 |  |  | 0.351 |  |  | 0.627 |
| **M0** | 128 (47.1%) | 140 (51.5%) |  | 131 (48.2%) | 137 (50.4%) |  | 136 (50%) | 132 (48.5%) |  | 127 (46.7%) | 141 (51.8%) |  | 124 (45.6%) | 144 (52.9%) |  |
| **M1** | 1 (0.4%) | 3 (1.1%) |  | 2 (0.7%) | 2 (0.7%) |  | 3 (1.1%) | 1 (0.4%) |  | 3 (1.1%) | 1 (0.4%) |  | 1 (0.4%) | 3 (1.1%) |  |
| **Gender, n (%)** |  |  | 0.825 |  |  | 1.000 |  |  | 0.377 |  |  | 1.000 |  |  | 0.122 |
| **Female** | 62 (16.6%) | 59 (15.8%) |  | 60 (16%) | 61 (16.3%) |  | 56 (15%) | 65 (17.4%) |  | 61 (16.3%) | 60 (16%) |  | 53 (14.2%) | 68 (18.2%) |  |
| **Male** | 125 (33.4%) | 128 (34.2%) |  | 127 (34%) | 126 (33.7%) |  | 131 (35%) | 122 (32.6%) |  | 126 (33.7%) | 127 (34%) |  | 134 (35.8%) | 119 (31.8%) |  |
| **Age, n (%)** |  |  | 1.000 |  |  | 1.000 |  |  | 0.277 |  |  | 0.797 |  |  | 0.277 |
| **<=60** | 89 (23.9%) | 88 (23.6%) |  | 89 (23.9%) | 88 (23.6%) |  | 83 (22.3%) | 94 (25.2%) |  | 87 (23.3%) | 90 (24.1%) |  | 83 (22.3%) | 94 (25.2%) |  |
| **>60** | 98 (26.3%) | 98 (26.3%) |  | 98 (26.3%) | 98 (26.3%) |  | 104 (27.9%) | 92 (24.7%) |  | 100 (26.8%) | 96 (25.7%) |  | 104 (27.9%) | 92 (24.7%) |  |
| **Vascular invasion, n (%)** |  |  | 0.404 |  |  | 0.285 |  |  | 0.676 |  |  | 0.241 |  |  | **0.029** |
| **No** | 110 (34.6%) | 98 (30.8%) |  | 111 (34.9%) | 97 (30.5%) |  | 105 (33%) | 103 (32.4%) |  | 116 (36.5%) | 92 (28.9%) |  | 117 (36.8%) | 91 (28.6%) |  |
| **Yes** | 52 (16.4%) | 58 (18.2%) |  | 51 (16%) | 59 (18.6%) |  | 59 (18.6%) | 51 (16%) |  | 53 (16.7%) | 57 (17.9%) |  | 47 (14.8%) | 63 (19.8%) |  |
| **Age, meidan (IQR)** | 61 (52, 69) | 61 (51.25, 68) | 0.732 | 61 (51.5, 69) | 61 (52, 68.75) | 0.961 | 62 (52, 68.5) | 60 (51, 69) | 0.448 | 62 (52, 69) | 61 (51, 68) | 0.617 | 62 (53, 69) | 60 (51, 68) | 0.316 |
